# Misuse of Cardiac Lipid upon Exposure to Toxic Trace Elements—A Focused Review

**DOI:** 10.3390/molecules27175657

**Published:** 2022-09-02

**Authors:** Kaviyarasi Renu, Anirban Goutam Mukherjee, Uddesh Ramesh Wanjari, Sathishkumar Vinayagam, Vishnu Priya Veeraraghavan, Balachandar Vellingiri, Alex George, Ricardo Lagoa, Kamaraj Sattu, Abhijit Dey, Abilash Valsala Gopalakrishnan

**Affiliations:** 1 Centre of Molecular Medicine and Diagnostics (COMManD), Department of Biochemistry, Saveetha Dental College & Hospitals, Saveetha Institute of Medical and Technical Sciences, Saveetha University, Chennai 600077, Tamil Nadu, India; 2 Department of Biomedical Sciences, School of BioSciences and Technology, Vellore Institute of Technology (VIT), Vellore 632014, Tamil Nadu, India; 3 Department of Biotechnology, PG Extension Centre, Periyar University, Dharmapuri 636701, Tamil Nadu, India; 4 Human Molecular Cytogenetics and Stem Cell Laboratory, Department of Human Genetics and Molecular Biology, Bharathiar University, Coimbatore 641046, Tamil Nadu, India; 5 Jubilee Centre for Medical Research, Jubilee Mission Medical College and Research Institute, Thrissur 680005, Kerala, India; 6 School of Technology and Management, Polytechnic Institute of Leiria, 2411-901 Leiria, Portugal; 7 Applied Molecular Biosciences Unit, NOVA University of Lisbon, 2819-516 Caparica, Portugal; 8 Department of Life Sciences, Presidency University, Kolkata 700073, West Bengal, India

**Keywords:** heavy metals, cadmium, arsenic, mercury, lead, lipotoxicity, cardiotoxicity

## Abstract

Heavy metals and metalloids like cadmium, arsenic, mercury, and lead are frequently found in the soil, water, food, and atmosphere; trace amounts can cause serious health issues to the human organism. These toxic trace elements (TTE) affect almost all the organs, mainly the heart, kidney, liver, lungs, and the nervous system, through increased free radical formation, DNA damage, lipid peroxidation, and protein sulfhydryl depletion. This work aims to advance our understanding of the mechanisms behind lipid accumulation via increased free fatty acid levels in circulation due to TTEs. The increased lipid level in the myocardium worsens the heart function. This dysregulation of the lipid metabolism leads to damage in the structure of the myocardium, inclusive fibrosis in cardiac tissue, myocyte apoptosis, and decreased contractility due to mitochondrial dysfunction. Additionally, it is discussed herein how exposure to cadmium decreases the heart rate, contractile tension, the conductivity of the atrioventricular node, and coronary flow rate. Arsenic may induce atherosclerosis by increasing platelet aggregation and reducing fibrinolysis, as exposure interferes with apolipoprotein (Apo) levels, resulting in the rise of the Apo-B/Apo-A1 ratio and an elevated risk of acute cardiovascular events. Concerning mercury and lead, these toxicants can cause hypertension, myocardial infarction, and carotid atherosclerosis, in association with the generation of free radicals and oxidative stress. This review offers a complete overview of the critical factors and biomarkers of lipid and TTE-induced cardiotoxicity useful for developing future protective interventions.

## 1. Toxic Trace Elements and Cardiotoxicity

Heavy metals and metalloids are potentially toxic to humans and animals, being able to cause deleterious effects even at low doses [1,2]. These toxic trace elements (TTE) are common in the soil, water, atmosphere, and food. However, small doses and acute or chronic exposure conditions can lead to serious health issues for the human organism. Human exposure occurs via environmental contamination, occupational exposure, and food accumulation [3,4,5]. The environmental contamination with TTEs is both natural and anthropogenic. It can affect almost all the organs, but mainly the heart, kidney, liver, lungs, and nervous system [6,7,8].

A list of the TTEs that are more hazardous is provided by the Toxic Substances and Disease Registry Priority List of Hazardous Substances [9]. The toxicity mechanisms of heavy metals and metalloids are diverse and include enzymatic inhibition, oxidative stress, and antioxidant metabolism impairment. Associated with free radical formation, TTEs cause DNA damage, lipid peroxidation, and oxidation of protein sulfhydryls [10].

The TTEs covered in this review are Cadmium (Cd), Arsenic (As, a metalloid), Mercury (Hg), and Lead (Pb), the best described to affect lipid metabolism. The augmented level of Cd causes cardiovascular diseases, hypertension, and hemorrhages [11]. Many pathological mechanisms give rise to cardiovascular diseases in response to Cd [12]. Cd causes vascular smooth muscle distress by augmenting phosphokinase levels [13]. Cd impairs the activity of potassium and sodium ATPase, and this inhibition causes elevated calcium (Ca) levels during hypertension in the smooth muscle. The main reasons for the cardiotoxicity are oxidative stress with attenuated antioxidant defenses and mitochondrial enrichment [14]. The cardiotoxicity of As is also mediated by increased oxidative stress with low antioxidant levels and mitochondrial enrichment [15].

Hg induces parasympathetic activity in the heart, which further causes heart rate variability. The main mechanisms Hg cardiotoxicity are the generation of free radicals, decreased antioxidant levels, and increased oxidative stress [16]. Hg favors hypertension, myocardial infarction, and carotid atherosclerosis. It induces the phospholipase A2, which catalyzes the hydrolysis of glycerol phospholipids and liberates arachidonic acid and lysophosphatidic acid. These catabolic products, such as prostaglandins and thromboxanes, lead to coronary heart diseases [17].

Pb can cause chronic illness in the heart. Different studies show that exposure to Pb causes cardiovascular diseases and hypertension [18,19]. The primary mechanism of Pb-induced cardiotoxicity was oxidative stress augmentation and impairment of the availability of nitric oxide (NO). Additionally, Pb causes endothelial dysfunction, leading to arteriosclerosis, thrombosis, and cardiovascular diseases [20].

## 2. Toxic Trace Elements Alter the Lipid Levels in Circulation and the Cardiovascular Risk

Exposure to Cd, Pb and Hg alters the level of blood lipids, such as total cholesterol (TC) and low-density lipoprotein cholesterol (LDL-C) [21,22]. Pb exposure alters the serum’s lipid and alpha-tocopherol levels [23]. The exposure to Cd augments the serum lipid profile via increasing triglycerides (TG) and LDL-C, and attenuates the high-density lipoproteins cholesterol (HDL-C) level. These changes are associated with increased lipid peroxidation and decreased reduced glutathione (GSH) levels [24].

Exposure to As reduces the HDL-C and the ratio of HDL-C/LDL-C, while it increases the levels of TG, TC, LDL-C, and electrolytes [25]. Exposure to Hg also augments cholesterol levels as LDL-C in serum [26]. Similarly, occupational exposure to Pb increased LDL-C and cholesterol in serum [27], leading to hyperlipidemia and cardiovascular disease risk. 

A recent work investigated the associations between blood levels of 10 metals and lipidomic profiles among 83 pregnant women from Puerto Rico [28]. A total of 587 lipids were measured in the plasma. The results showed strong negative associations of manganese and zinc with plasmenyl-phosphatidylethanolamine (PLPE), particularly those containing polyunsaturated fatty acid chains. In contrast, exposure to As and Hg was positively associated with PLPE and plasmenyl-phosphatidylcholine [28].

## 3. Lipid Misuse and Cardiotoxicity

The heart is an organ that needs high energy for its function via fatty acids. Among all the substrates, more than 70% are utilized to produce energy, such as ATP [29]. The regulation of fatty acid uptake and oxidation prevents the accumulation of lipids in the heart. During metabolic syndrome or exposure to heavy metals, lipid accumulation is favored by increased free fatty acid (FFA) levels in the circulation [30,31]. 

The increased level of lipid in the myocardium threatens the heart’s function. This dysregulation of the lipid metabolism can cause damage to the structure of the myocardium, including fibrosis in cardiac tissue, apoptosis of myocytes, and decreased contractility due to mitochondrial dysfunction. Accumulating excess lipids in the heart leads to lipotoxicity, associated with cardiomyopathy and cardiovascular dysfunction [29,32].

## 4. Epidemiological Evidence of Lipid Alterations after Exposure to Toxic Trace Elements

Exposure to heavy metals increases the risk of cardiovascular illness in the Korean population [33]. Human exposure to Cd increased blood lipids, such as TC, augmented TG, LDL-C, and decreased HDL-C, in close association with the blood Cd level in 1489 workers exposed to the metal in China. These alterations substantiate the condition of dyslipidemia [34]. The increased Cd level in urine was associated with augmented cardiovascular mortality in a population of 3348 individuals between the age of 45–74 years (American Indian) [35].

The exposure of 57 workers to As showed that there is an increased level of the lipoprotein (a) (LP(a)), a ratio of apolipoprotein-B (Apo-B)/apolipoprotein-A1 (Apo-A1) and Apo-B level and a decreased level of Apo-A1, indicative of high-risk of heart diseases [36]. The exposure to Hg workers that experienced from Korean National Environmental Health Survey cycle 3 in 3228 samples indicated an increase in TC, TG, LDL, and VLDL [37]. Exposure to Pb of battery workers in Lucknow city (India) saw increased TC/HDL-C and LDL/HDL ratios [38]. The exposure to Pb in Abeokuta (Nigeria) was also associated with an altered lipid profile and increased risk of cardiovascular diseases [39]. In a different approach, exposure to Ni was associated with coronary heart disease in aorta patients [40]. Overall, the epidemiological data here discussed underlines the contribution of exposures to TTEs for dyslipidemia and increased risk of cardiovascular illness.

## 5. Cadmium-Associated Cardiotoxicity via Lipid Metabolism

It has been demonstrated that Cd induces physiological and biochemical changes to the heart. Exposure to Cd in the form of CdCl_2_ decreases the heart rate, contractile tension, the conductivity of the atrioventricular node, and coronary flow rate [41,42]. The toxicity of Cd is associated with the homeostatic cardiac repair mechanism and metabolism at a cellular level, which result in the generation of reactive oxygen species (ROS) and apoptosis [43].

Of note, low doses of Cd in the water administered to mice increased oxidative stress markers and affected mitochondrial respiration and lipid metabolism in the lungs [44]. Significant increases in the expression of pro-inflammatory genes (NF-kB and COX-1) were also described in the liver and kidney of mice treated with cadmium chloride [8].

**Figure 1:**
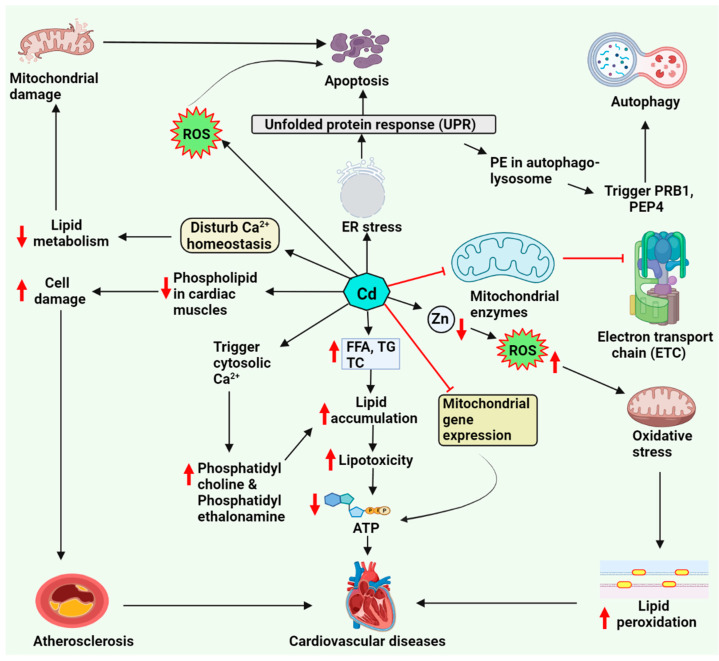
Cadmium-induced lipid deregulation and cardiotoxicity. Cadmium generates ROS, resulting in oxidative stress, which leads to increased lipid peroxidation; it also causes endoplasmic reticulum stress and disturbs calcium signaling. These basic mechanisms alter lipid metabolism and cause mitochondrial damage, leading to cardiac cell apoptosis and cardiovascular damage. Abbreviations: ATP, adenosine triphosphate; FFA, free fatty acid; PE, phosphatidylethanolamine; PEP4, pepstatin A; PRB1, proteinase B1; ROS, reactive oxygen species; TC, total cholesterol; TG, triglyceride.

Cd reduces the expression of mitochondrial respiration genes and the production of ATP in rat hearts [45]. Additionally, Cd inhibits the mitochondrial electron transport chain’s four complexes (complex I–IV) [46]. Moreover, Cd decreases the phospholipid level in the cardiac muscle, damaging the cells’ membrane [41]. Figure 1 illustrates the putative mechanisms of cadmium-induced lipotoxicity and cardiotoxicity.

### 5.1. Cardiac Lipotoxicity upon Cadmium Exposure

Cardiac lipotoxicity is present in metabolic disorders such as diabetes and heart diseases. The heart function highly depends on the capacity of the metabolism. Cd increased the weight of the heart in a rat model [47]. Similarly, another study showed that toxic chemicals increase organ weight due to the accumulation of lipids and inflammation [14]. The FFA, TC, and TG levels are increased in the Cd-exposed heart, and in a dose-dependent manner. During diabetic cardiomyopathy, there is an elevated FFA level which contributes to lipid accumulation in the heart. Also, the unbalance between the fatty acid uptake and its oxidation leads to TG accumulation, such as cardiac lipotoxicity, and decreases ATP in the heart, potentially leading to cardiac damage [47].

Similarly, the Cd-induced deregulation of lipid levels in the heart is associated with decreased energy levels in the myocardium, further leading to cell death [14]. In convergence, Cd inhibited mitochondrial fatty acid oxidation and caused hepatic mitochondrial dysfunction and fatty acid oxidation deficiency in a chronic exposure mouse model. On this basis, we can hypothesize that Cd causes lipid accumulation and oxidative damage via deregulating Zn, and further causes cardiac lipotoxicity.

### 5.2. Role of Zinc and Calcium Homeostasis in Lipid Accumulation

Cd has some physical and chemical properties comparable to zinc (Zn). Exposure to Cd depletes the intracellular level of Zn and causes adverse side effects in different organs [48]. The depletion of Zn disturbs the metabolism and function of lipids. Except for the F2-IsoP, where Zn offered only partial protection, supplementation with Zn prevented Cd-induced alterations in lipid metabolism in a study with male Wistar rats administered 5 and 50 mg Cd/L [49]. According to the findings, Zn supplementation during Cd exposure may have a protective effect on lipid metabolism, including the potential to prevent hyperlipidemia, particularly hypercholesterolemia, and protect against lipid peroxidation [49,50]. This needs to be further elucidated in other models and humans.

Zn has two plasma membrane transporters, Zrt1p, which has a high affinity for Zn, and Zrt2p, which has a low affinity for Zn and is activated by the Zn-responsive transcription factor ZAP1. Zrt1p also transports Cd in yeast [51]. This transporter depends on ZAP1 activity and protects from Cd toxicity via decreasing lipid accumulation (TAG) and oxidative damage through ROS. These observations indicate that Cd causes lipid accumulation and oxidative damage disturbances by deregulating Zn homeostasis [52].

The high exposure to Cd was described as reducing calcium intake by cardiomyocytes [53]. Cd alters the calcium homeostasis, causing ROS production and mitochondrial damage that disturbs lipid metabolism and favor apoptosis. On the other hand, it activates unfolded protein response (UPR) through endoplasmic reticulum (ER) stress that can further contribute to apoptosis [54]. Activated UPR mediates phosphatidylethanolamine in the autophagosomes and can trigger autophagyby involving proteases indicated in Figure 1 [54,55]. Cd inhibits the ER Ca^2+^ ATPase SPF1, thereby increasing the cytosolic calcium and lipid droplet (LD) accumulation [56].

Altogether, there is experimental evidence for the disturbance of Ca and Zn homeostasis and related cellular signaling processes under conditions of Cd exposure [57]. These changes can be implicated in lipid accumulation. The hypothetical role in cardiac lipotoxicity needs further elucidation in animal models and humans.

### 5.3. Lipid-Driven Signaling Pathways upon Cadmium Exposure

Exposure to Cd causes ER stress and leads to cell death via impairment of energy metabolism. Cd impairs the energy metabolism via AKT/mTOR pathway inhibition [58]. The cardiomyocytes are highly dependent on the energy metabolism for function, which becomes impaired by Cd. The decreased energy metabolism defeats the cardiomyocyte structure and function, leading to cellular stress and further causing cell death. In cardiomyocytes, exposure to Cd concentrations 1 and 100 μM for 4 to 15 h reduces the energy production via ER stress by deregulating respiratory gene expressions in mitochondria [45]. This impairment of the mitochondrial gene expression and reduced ATP is attained via AKT/mTOR pathway. Consequently, ER stress and decreased energy production lead to cell death in cardiomyocytes upon cadmium exposure [45]. Cd exposure increases the TG level and synthesis of LD indifferent strains of yeast cells, apparently being implicated in the increased expression of an ER fatty acid desaturase essential for the synthesis of monounsaturated fatty acids [52].

## 6. Arsenic-Associated Cardiotoxicity via Lipid Metabolism

The toxicokinetics of As is influenced by the conversion of inorganic As(III) into mono- and di-methylated metabolites [59]. The synthesis of methylated metabolites decreased the susceptibility to As by accelerating the rate of clearance; in other words, methylation is accepted to favor the detoxification of As [60]. More recently, the methylated metabolites have also been implicated in the development of certain harmful effects of As poisoning, pointing methylation a role in activation of the toxicant [61]. The As methylation is enzyme-catalyzed, and there is a significant interindividual variability in As metabolism, which affects the metalloid’s activation and detoxification [62] and, therefore, the susceptibility to As [63].

**Figure 2:**
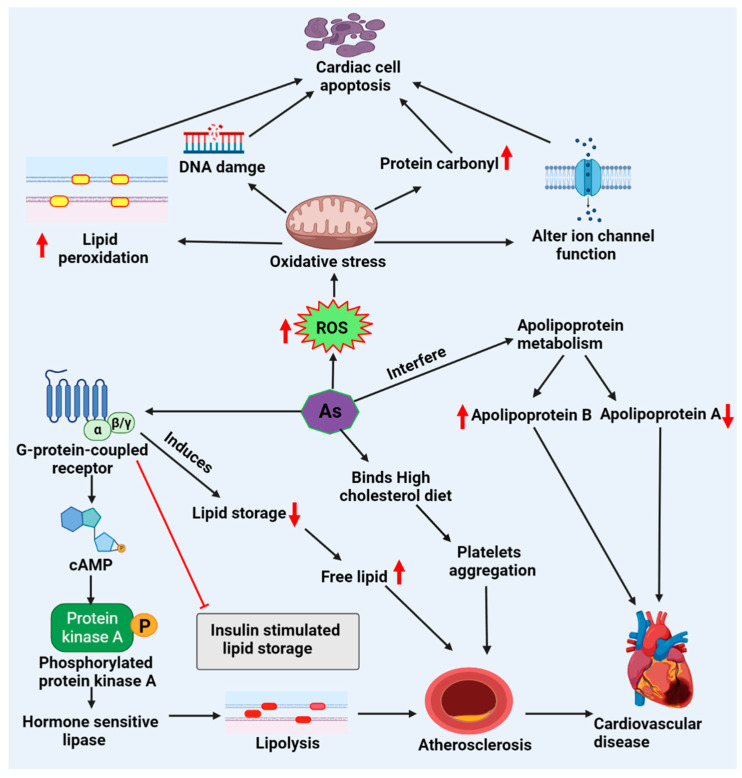
Arsenic-induced lipid deregulation and cardiotoxicity. Arsenic generates ROS, resulting in oxidative stress, which causes DNA damage, alters ion channel function, and increases lipid peroxidation, disturbing the mitochondrial process in cardiac cell apoptosis and cardiotoxicity. Moreover, arsenic activates the process of lipolysis via G-protein-coupled receptors by activating hormone-sensitive lipase, decreasing lipid storage, and increasing free lipid, further promoting atherosclerosis and cardiovascular diseases. Abbreviations: cAMP, cyclic adenosine monophosphate; ROS, reactive oxygen species.

As (III) induces oxidative stress, DNA fragmentation, apoptosis, and ion channel functional alterations lead to cardiotoxicity and decrease total thiol levels while raising oxidized glutathione (GSSG), lipid peroxidation, and protein carbonyl levels [64,65]. Indeed, disrupting the glutathione and mitochondrial redox systems, leading to DNA damage and lipid peroxidation, seem to be hallmarks of As toxicity [44]. Studies have revealed that As attaches to the SH-group of GSH or proteins with high cysteine content, producing ROS and playing a central role in the development of cardiovascular disorders (Figure 2) [66,67].

As exposure in rats causes atherosclerosis via platelet aggregation and attenuated fibrinolysis [68]. Chronic exposure to As increases the expression of TNF-α, interleukin(IL)-1, vascular endothelial growth factor, and vascular cell adhesion molecule, leading to cardiovascular diseases [66,67]. The early combination of As and high cholesterol diet decreases the HDL-C/LDL-C ratio without changing the TC level. This fall coincided with an elevated HSP 70 level [55]. These findings suggest that As-induced atherosclerosis is linked to the ratio of the two forms of cholesterol rather than a high TC level [69]. Alone or combined with a high cholesterol diet, As causes atherosclerosis by inducing transitory elevations in HSP 70 and hs-CRP [66,67]. Early combination exposure reduced the HDL-C/LDL-C ratio without affecting total cholesterol or triglyceride levels [70]. CETP-1 and LXR were inhibited, with the early combined exposure having the most significant effect. The inflammatory markers of atherosclerosis are HSP 70 and hs-CRP rose, which could be linked to the establishment of atherosclerosis [67,69,71,72].

### 6.1. Cardiac Lipotoxicity Evidence upon Arsenic Exposure

As and its conjugates have been shown in several studies to negatively impact systems linked to cardiomyopathy disorders, including insulin secretion and signaling, systemic inflammation, complement activation, lipid metabolism, and atherosclerosis. Changes in inflammatory markers and expression of the microRNA-21 closely related to cancer and inflammation have been associated with As exposure [44]. In addition, different studies indicate that As or its conjugates may affect lipid metabolism and cause pro-inflammatory reactions [36,67,73]. It has been revealed that As exposure can interfere with apolipoprotein metabolism, resulting in a rise in Apo-B and Apo-B/Apo-A1 ratios, a decrease in Apo-A1, and an elevated risk of acute cardiovascular disease events like myocardial infarction and acute cerebral strokes [36].

Humans can be predisposed to cardiovascular disorders such as hypertension, stroke, atherosclerosis, and Blackfoot disease if exposed to As over an extended period [74]. ROS causes oxidative damage that can impact gene expression, inflammatory responses, and nitric oxide homeostasis, which are major factors in As-induced toxicity [75]. As in drinking water can cause vascular endothelial dysfunction, manifesting as a mismatch between vascular relaxation and contraction. These consequences, in turn, raise the risk of vascular disorders like hypertension and atherosclerosis [66,76]. The endothelium is activated by oxidized low-density lipoprotein (oxLDL). The active endothelium produces cell adhesion molecules, which attract monocytes, transmigrating the subendothelial region and differentiating [72]. The macrophages subsequently take up the oxLDL via scavenger receptors, resulting in the production of foam cells. The migration of medial smooth muscle cells into the intima characterizes the progression of fatty streaks to more complex lesions.

Smooth muscle cells in the intima multiply and suck up oxLDL, forming foam cells, synthesizing extracellular matrix proteins, and forming a fibrous cap [72]. In a study with 959 subjects, exposure to As was associated with carotid intima-media thickness, and the association was stronger in the case of lower As methylation capacity [77]. The As-promoted atherosclerosis was attributed to changes in cholesterol transport and metabolism [67]. The liver X receptors (LXR) are nuclear receptors with antiatherogenic action, promoting cholesterol efflux and inhibiting inflammation. Interestingly, the deletion of LXRα prevented As-enhanced plaque formation in mice, ascribable to inhibition of the receptor. However, the As-induced changes in-plaque composition seem to involve LXRα-independent mechanisms [78]. Very recently, metabolomics revealed that fatty acids and steroids accumulated in macrophages exposed to benzo[a]pyrene, and the changes in fatty acid metabolism elicited by this toxicant have been connected to aryl hydrocarbon receptor (AHR) signaling [79].

### 6.2. Lipid-Driven Signaling Pathways upon Arsenic Exposure

As suppresses adipocyte differentiation and enhances insulin resistance; however, little is known about the effects of As on adipose lipid storage and lipolysis. Increased cAMP levels are not associated with As-stimulated lipolysis [80]. G-protein-coupled receptors (GPCRs) may participate in the metabolic disorders induced by environmental As exposures, causing abnormal lipid accumulation and metabolism [81]. Excess lipid accumulation induces hypertrophic expansion of adipose tissue, inhibiting adipose regeneration and producing ectopic lipid storage in the liver, heart, and skeletal muscle [80,81,82].

The GPCR-mediated (e.g., β-adrenergic stimulation) increase in intracellular cAMP levels that induce protein kinase A (PKA), phosphorylation of hormone-sensitive lipase and PLIN1 is the canonical pathway for physiological lipolysis [83]. These signals enable activated lipase to access stored lipid droplet triglycerides to cleave lipids [83,84].

As (III) elicits epididymal adipose tissue remodeling, including enhanced adipocyte size and ectopic lipid deposition in muscle. In contrast, As (III) suppresses PLIN1 expression in vivo and cultured cells, resulting in increased lipolysis and reduced adipocyte lipid storage capacity. Increased lipolysis is found in various pathologic circumstances when PLIN1 expression is reduced. Reduced PLIN1 expression increases basal lipolysis but reduces triggered lipolysis. There is a PLIN1-dependent shift in basal lipolysis, which shows a new balanced state of lipolysis in the adipocytes upon As exposure [81,84].

Sphingosine 1-phosphate receptors (S1PR1/S1PR2) are another GPCR that regulates adipocyte activity and lipid storage [85]. S1PR1 has been demonstrated to mediate oxidant generation and remodeling in vascular endothelial cells in response to As (III) [86]. The effects of As (III) on lipid storage and lipolysis and the probable molecular processes underpinning these harmful metabolic effects have not been studied [80].

## 7. Mercury-Associated Cardiotoxicity via Lipid Metabolism

**Figure 3:**
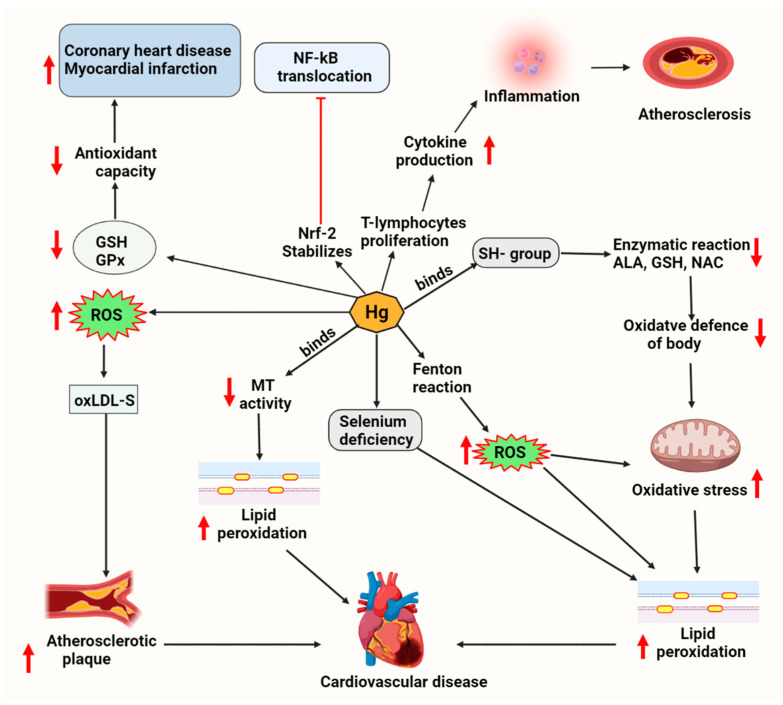
Mercury-induced lipid deregulation and cardiotoxicity. Mercury generates ROS through the Fenton reaction, which increases oxidative stress in the cell, increases lipid peroxidation, and causes metallothionein and selenium deficiency. Mercury compounds also decrease antioxidant levels and further causes inflammation, leading to cardiovascular dysfunction. Abbreviations: ALA, alpha-lipoic acid; GPx, Glutathione peroxidase; GSH, reduced glutathione; MT, metallothionein; NAC, N-acetylcysteine; Nfr-2, nuclear factor erythroid 2–related factor 2; NF-kB, nuclear factor-kappa B; oxLDL, oxidized low-density lipoprotein; ROS, reactive oxygen species; SH, sulfhydryl group.

Humans are exposed to Hg in different forms, such as methylmercury and elemental mercury vapor [87]. Hg has a strong affinity for sulfhydryl (-SH) groups, which deactivates a wide range of enzymatic reactions, amino acids, and sulfur-containing antioxidants [GSH, N-acetyl cysteine (NAC), alpha-lipoic acid (ALA)], resulting in a reduction in oxidant defense and an increase in oxidative stress [88]. Methylmercury causes mitochondrial dysfunction by lowering ATP levels, lowering glutathione levels, and increasing lipid peroxidation (Figure 3) [89]. Low micromolar concentrations of Hg(II) were enough to cause substantial increases in ROS, disturb the thioredoxin and glutathione/glutaredoxin systems, and induce the production of pro-inflammatory cytokines by different cells [87,90].

### 7.1. Cardiac Lipotoxicity Evidence upon Mercury Exposure

Hg is one of the critical risk factors for atherosclerosis. Exposure to the metal causes an overproduction of ROS, resulting in the oxidation of low-density lipoprotein (oxLDL) cholesterol, which can contribute to the onset and progression of atherosclerotic plaques [91].

The affinity of Hg for sulfhydryl groups, which make up much of the antioxidant capacity of plasma, such as glutathione, NAC, and ALA, decreases membrane and plasma antioxidant defense [89,91]. Hg acts as a direct and indirect catalyst in Fenton-type reactions, increasing the generation of superoxide anions and other ROS [89]. Hg–selenium insoluble complexes limit selenium availability, which is required for glutathione peroxidase (GPx) activity, which breaks down hydrogen peroxides and other toxic peroxidation products. Reduced GPx and low GSH levels have been linked to a higher risk of coronary heart disease and myocardial infarction. Since GSH levels fall, plasma and intracellular antioxidant capability are lowered [88,89]. Emphasizing the relevance of these molecular mechanisms, NAC and selenium/selenite supplementation showed an ability to increase GSH and attenuate inflammatory responses to Hg and As [87,92].

The progression of cardiovascular disease, particularly atherosclerosis, is aided by an increase in ROS and a decrease in antioxidant activity [93,94]. Lipid peroxidation, an autocatalytic process, is induced by free radicals [91]. Polyunsaturated fatty acids in cell membranes, such as phospholipids in LDL, decrease through a chain reaction during lipid peroxidation. Monocytes are drawn into the vascular endothelium by oxLDL, transforming into macrophages. In the endothelium of vessels, macrophages eliminate ox-LDL particles and deposit intracellular lipids. The macrophages produce pro-inflammatory cytokines, leading to monocyte recruitment and foam cell proliferation. These cells cause atherosclerotic lesions to form in the early stages of the disease [94,95]. Studies with macrophages indicated that inorganic Hg inhibits NO production and promotes the expression of pro-inflammatory TNF-α and IL-6 [90].

### 7.2. Lipid-Driven Signaling Pathways upon Mercury Exposure

Inflammation plays a significant role in Hg toxicity [96]. At nontoxic levels, Hg enhances T lymphocyte proliferation and cytokine production [97]. Hg has been demonstrated to deactivate nuclear factor-kB (NF-kB), a crucial transcription factor that regulates the expression of inflammatory and immune-related genes [6,90]. 

Upon pro-inflammatory cytokines stimulation, IkB is phosphorylated, causing NF-kB activation and transport to the nucleus, binding to DNA. TNF-α production is then triggered, potentiating an inflammatory response [98]. Excessive oxidative stress causes NF-kB activation [99], but oxidants are also important to maintain or trigger the nuclear factor erythroid 2-related factor 2 (Nrf2) pathway [44]. The Nrf2 signaling is an antioxidant defense pathway that modulates the expression of several antioxidant enzymes like GPx, catalase and superoxide dismutase (SOD). Under healthy conditions, Kelch-like ECH-associated protein 1 (Keap1) conserves Nrf2 in the cytoplasm, rendering it inactive [100]. Nrf2 is released from Keap1 and translocates into the nucleus in cells activated by ROS [101]. HgCl_2_ exposure caused NF-kB nuclear translocation, which elevated TNF-α protein levels, although Nrf2 preventd NF-kB nuclear translocation. The inactivation of NF-kB was attributed to Hg attaching onto sulfhydryl groups [102], a mechanism that probably also explains Hg inhibition of NF-kB activation in macrophages under inflammatory conditions [90].

More recently, p38 activation by Hg(II) has been highlighted [87,90], and p38-mediated activation of peroxisome proliferator-activated receptors (PPARs) participates in the regulation of lipid metabolism [103].

## 8. Lead-Associated Cardiotoxicity via Lipid Metabolism

Promotion of lipid peroxidation and impairment of the activity of blood SOD is often found to be associated with lead exposure [6]. Lipid peroxidation is found to be directly related to damage to the membrane tissues. It is found to be a risk factor for several vascular diseases. Upregulated lipid peroxidation poses severe threats to blood clotting. It leads to vascular endothelium damage, which gives us an idea of the mechanism of Pb-induced atherosclerosis [104]. Exposure to Pb generates peroxynitrite (ONOO-), which causes NO deficiency and severe cardiovascular complications [20]. The scarcity of physiologically active NO is directly related to oxidative stress. Adding to this exhaustion of the NOS cofactor tetrahydrobiopterin, avid seizure of NO by ROS, and uncoupling of eNOS can diminish NO availability under oxidative stress [105,106]. A significant compensatory raise of Mn-SOD was reported in the lead-induced hypertension patients’ hearts.

**Figure 4:**
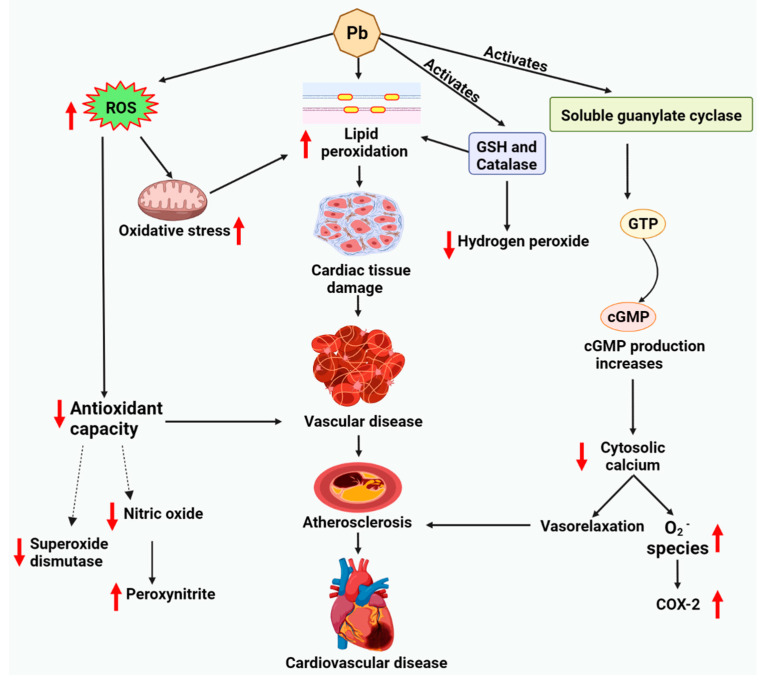
Lead-induced lipid deregulation via cardiac tissue damage and cardiotoxicity. Lead generates ROS, resulting in oxidative stress on the cell and decreasing the antioxidant and cytosolic calcium levels. Lead exposure also upregulates lipid peroxidation implicated in the cardiotoxicity process. Abbreviations: cGMP, cyclic guanosine monophosphate; COX, cyclooxygenase; GSH, reduced glutathione; GTP, guanosine-5’-triphosphate; ROS, reactive oxygen species.

However, no considerable change is observed concerning catalase or GPx [20]. Since catalase and GPx are accountable for tumbling H_2_O_2_ and lipoperoxides, a deficiency of a proper upsurge in their tissue levels can aggravate lead-induced oxidative stress. The instigation of soluble guanylate cyclase (sGC), which marks the generation of the second messenger cGMP from GTP, is accountable for most of NO’s biological effects [107]. cGMP promotes vasorelaxation by plummeting cytosolic Ca concentration in vascular smooth muscle cells (VSMCs) via Ca confiscation in the sarcoplasmic reticulum and inhibition of calcium entry [108]. Concentration-dependent downregulation of sGC expression, the promotion of superoxide generation, and overexpression of cyclooxygenase-2 (COX-2) arise in the presence of Pb (Figure 4) [20,106].

### 8.1. Cardiac Lipotoxicity Evidence upon Lead Exposure

Only a limited number of studies have established the connection between Pb exposure and cardiovascular diseases, including coronary artery disease, stroke, and peripheral arterial disease. Dyslipidaemia has been recognized as a menace for cardiac ailment in the African population and an upsurge in deaths in industrialized and unindustrialized rural areas [109,110]. Even though black Africans have lower cholesterol levels than at northern European countries [111], this study showed that Pb exposure alters cholesterol breakdown and increases the risk of vascular illness and atherosclerosis in Pb-exposed subjects. Research by different authors showed that, in the presence of hemoglobin or Fe^2+^, Pb promotes lipid oxidation [112,113]. Liposomes, erythrocytes, microsomal fractions, and rat brain homogenates promote Fe^2+^-initiated lipid oxidation [114]. The exact pathophysiological mechanisms behind these Pb-induced variations are not entirely acknowledged.

### 8.2. Lipid-Driven Signaling Pathways upon Lead Exposure

In the standard process of oxygen metabolism, considerable quantities of ROS, such as superoxide (O_2_^−^) and H_2_O_2_, are formed and firmly reserved by the antioxidant defense system. However, oxidative stress is produced by various pathophysiological settings. Uncontrolled ROS root tissue injury occurs in the existence of oxidative stress by unswervingly destroying and denaturing functional/structural components and triggering redox-sensitive transcription factors and signal transduction paths. Oxidative stress influences the progression of many acute and chronic pathologies, including hypertension and cardiovascular disease, which are affected by lead exposure [115,116,117]. By participating in Fenton- and Haber-Weiss-type reactions, Pb^2+^ can increase ROS generation and oxidative stress. This effect was documented in vivo, in vascular tissues, and in vitro with endothelial cells and VSMC [20,118].

## 9. Conclusions

This review provides an overall outlook on the cardiotoxicity of several TTEs. Cd, As, Hg and Pb are the most common metals and metalloids inducing cardiovascular toxicity. These TTEs cause oxidative stress via increased lipid peroxidation and energy metabolism impairment by inhibiting the mitochondrial enzyme complexes in the electron transport chain, resulting in ER stress and eventually cell death. Cd and Pb can also trigger alterations in cellular calcium homeostasis, a central player in regulating metabolism and many other functions in endothelial and muscular cells. 

Different mechanisms involved in lipid accumulation lead to misuse of cardiac lipids upon TTEs exposure. Exposure to Cd increases FFA and lipid accumulation, which causes lipotoxicity via decreased ATP, and leads to cardiovascular diseases. As exposure causes platelet aggregation, activates lipolysis, decreases lipid storage, and increases FFA, promoting atherosclerosis. Exposure to Hg and Pb is also closely associated with oxidative stress-mediated inflammation and atherosclerosis, although there is no data on the effect on lipid metabolism and levels in circulation. There is also evidence of important roles played by metal-induced alterations of some signaling routes, like AKT/mTOR and NF-kB pathways. 

Nevertheless, more research is needed in the future to determine the potential effects of TTEs on signaling pathways like those involving PPAR-α, PPAR-γ coactivator-1α, and AHR in lipid metabolism and lipotoxicity. Moreover, advanced lipidomics and live-cell imaging techniques are warranted to provide a more comprehensive understanding of the changes in the lipids in circulation and lipid droplet dynamics, lipid homeostasis and lipotoxicity in cells.

## Data Availability

Data are available from the authors on request (K.R.; R.L.; A.V.G.).
